# Biphasic oxygen tension promotes the formation of transferable blastocysts in patients without euploid embryos in previous monophasic oxygen cycles

**DOI:** 10.1038/s41598-023-31472-4

**Published:** 2023-03-15

**Authors:** Hsiu-Hui Chen, Chun-I Lee, Chun-Chia Huang, En-Hui Cheng, Tsung-Hsien Lee, Pin Yao Lin, Chien-Hong Chen, Maw-Sheng Lee

**Affiliations:** 1 Division of Infertility, Lee Women’s Hospital, Taichung, Taiwan; 2 Institute of Medicine, Chung Shan Medical University, Taichung, Taiwan; 3 Department of Obstetrics and Gynecology, Chung Shan Medical University Hospital, Taichung, Taiwan; 4 Department of Medical Research, Chung Shan Medical University Hospital, Taichung, Taiwan; 5 Post Baccalaureate Medicine, National Chung-Hsing University, Taichung, Taiwan

**Keywords:** Embryology, Reproductive biology

## Abstract

This study evaluated whether the concentration of biphasic O_2_ (5–2%) promotes the formation of qualified blastocysts (QBs) and euploid blastocysts and the probability of cycles with transferable blastocysts. The paired experimental design included a total 90 patients (180 cycles) without euploid blastocysts in previous monophasic O_2_ (5%) cycles were enrolled for an additional cycle of biphasic O_2_ (5–2%). In the biphasic O_2_ (5–2%) group, the QB rate (35.8%, 225/628) was significantly higher than that in the monophasic O_2_ (5%) group (23.5%, 137/582; *p* < 0.001). In addition, the euploid blastocyst number (0.5 ± 0.8) and the percentage of cycles with transferable blastocysts were significantly higher in the biphasic O_2_ (5–2%) group (57.8%, 52/90) than those in the monophasic O_2_ (5%) group (0 and 35.6%, 32/90, respectively; *p* < 0.01). Multivariable regression analysis also indicated that the QB rate and the probability of cycles with transferable blastocysts correlated with O_2_ tension (OR 1.535, 95% CI 1.325–1.777, and OR 3.191, 95% CI 1.638–5.679, respectively; *p* < 0.001). Biphasic O_2_ culture can be used as an alternative strategy to increase the euploid QBs and the probability of cycles with transferable blastocysts in patients with a poor prognosis.

## Introduction

Recent evidence indicates that the preimplantation embryo of most mammals develops in an increasingly hypoxic and dynamic O_2_ tension environment (2–8%) in vivo^1–3^. Although the majority of in vitro fertilization (IVF) centers have recognized the superiority of monophasic O_2_ (5%) tension in in vitro blastocyst development and intracellular redox balance, the biphasic O_2_ (5–2%) concentration in blastocyst culture systems remains more similar to the increasingly hypoxic and dynamic O_2_ tension environment in vivo^4–7^. According to previous research, human embryos in vivo usually cross the utero-tubal junction on day 3^8^; thus, the O_2_ tension is reduced from 5 to 7% in the oviduct to 1–5% in the uterus^2,8–10^. Therefore, for blastocyst development in vitro after day 3 of culture, O_2_ tension may need to be reduced from 5 to 2% to mimic the physiologic O_2_ tension dynamics in vivo^6^. According to a recent study, biphasic O_2_ (5–2%) has beneficial effects on pre-and peri-implantation embryonic development in mice^11^; biphasic O_2_ may be appropriate for human blastocyst formation^6^. In addition, biphasic O_2_ (5–2%) tension culture may be advantageous for patients with poor embryonic development by increasing the number of available high-quality blastocysts^12^.

According to previous studies, reducing O_2_ tension in the culture environment may reduce the levels of reactive oxygen species (ROS) and apoptosis, thereby promoting the in vitro development of embryos^5,6^. In addition to its effect on the quality of embryos, the O_2_ concentration may be linked to the mitochondrial structure^13^ and chromosomal errors^14,15^. ROS-induced damage increases the incidence of chromosome aneuploidy in mouse embryos obtained through IVF, and this damage is primarily mediated by chromosome missegregation^16^. The embryo culture system is another factor associated with ploidy during embryonic development, and the rate of embryonic aneuploidy may be related to the culture conditions of oocyte donor cycles in IVF laboratories^17^. Numerous factors, including culture medium (pH and constituents), light, temperature, and gas phase, may affect the quality of in vitro cultures, resulting in a substantial increase in the concentration of ROS^18^. Thus, during in vitro cultures, oxidative stress induced DNA damage is inevitable, disrupting the development of embryonic cells^19,20^. Aneuploidy is the leading cause of embryonic development arrest^21^. However, compared with high O_2_ (20%) tension conditions, low O_2_ (5%) conditions generate a larger number of high-quality embryos and euploid blastocysts available for embryo transfer^22^.

In humans, the effect of biphasic O_2_ (5–2%) on embryonic ploidy remains unknown. Similarly, whether biphasic O_2_ (5–2%) conditions are useful for embryonic ploidy remains unclear. In preimplantation genetic testing for aneuploidy (PGT-A) cycles, selecting euploid embryos for transfer may eventually increase the probability of embryonic implantation^23,24^. However, the success rate of cycles with PGT-A remains limited by the high incidence of cycles with unsuccessful PGT-A or cycles without transferrable euploid embryos^25^. Previous studies have reported the no-euploid-embryo rate of 25% for transfer^26^. However, the factors associated with the formation of embryos with an euploidy in humans vary greatly^27,28^. Even though oocytes are collected from young patients, such as oocyte donors^29^ or young patients (22–25 years) within fertility^30^, the aneuploidy rate in some IVF cycles seems to be high. Therefore, this study enrolled patients without euploid blastocysts for transfer in previous monophasic O_2_ (5%) cycles for determining whether the use of biphasic O_2_ (5–2%) in the next culture cycle can promote the formation of qualified blastocysts (QBs) and euploid blastocysts. In addition, improvements in cycles with at least one euploid blastocyst or with at least one transferable blastocyst (non-aneuploidy) were also compared between the monophasic O_2_ (5%) and the biphasic O_2_ (5–2%) groups. Our primary goal was to examine the formation of euploid blastocyst in two O_2_ tension groups. Our secondary goal was to determine the rate of QBs formation, transferable blastocysts and the probability of cycles with transferable blastocysts in the two O_2_ tension groups.

## Results

### Differences in baseline characteristics between biphasic O_2_ (5–2%) and monophasic O_2_ (5%) groups

Table 1 lists the baseline characteristics of the monophasic O_2_ (5%) group (*n* = 90) and biphasic O_2_ (5–2%) group (*n* = 90). The average age of the women included in this study was 39.5 ± 3.7 (27–45) years, and their average anti-Müllerian hormone (AMH) level and body mass index (BMI) were 2.3 ± 1.8 (0.5–13.7) ng/mL and 22.9 ± 3.8 (17.6–36.1) kg/m^2^, respectively. Paired comparisons revealed no significant differences in the type of ovarian stimulation protocol, the dosage of FSH, the level of E_2_ on the day of hCG injection, the level of P_4_ on the day of hCG injection, the numbers of MII oocytes, and the numbers of 2PN between the groups. However, the level of luteinizing hormone (LH) on the day of human chorionic gonadotropin (hCG) injection was significantly higher in the monophasic O_2_ (5%) group (2.6 ± 2.5 IU/L) than in the biphasic O_2_ (5–2%) group (1.7 ± 1.7 IU/L) (*p* = 0.001). In addition, the average number of oocytes was significantly smaller in the monophasic O_2_ (5%) group (10.7 ± 4.7) than in the biphasic O_2_ (5–2%) group (11.5 ± 5.9) (*p* = 0.047).

**Table 1:** Baseline characteristics of biphasic O_2_ (5–2%) and monophasic O_2_ (5%) concentration groups.

Characteristics	Monophasic (5%) O_2_	Biphasic (5–2%) O_2_	*p* value
OPU cycles (patients)	90	90	–
*Ovarian stimulation*			
GnRH agonist (%)	15.6 (14/90)	22.4 (20/90)	0.245
GnRH antagonist (%)	85.0 (76/90)	77.6 (70/90)	0.203
Total dose of FSH (IU, mean ± SD)	3015 ± 556 (2100–4575)	3117 ± 641 (1200–5775)	0.126
E_2_ levels (pg/mL, mean ± SD)	1562 ± 1180 (141–8763)	1423 ± 862 (104–4510)	0.619
P_4_ levels (ng/mL, mean ± SD)	0.84 ± 0.48 (0.13–2.66)	0.78 ± 0.55 (0.15–3.89)	0.340
LH levels (IU/L, mean ± SD)	2.6 ± 2.5 (0.2–17.3)	1.7 ± 1.7 (0.2–10.3)	0.001
Numbers of retrieved oocytes (mean ± SD)	10.7 ± 4.7 (2–24)	11.5 ± 5.9 (3–33)	0.047
Numbers of mature oocytes (MII) (mean ± SD)	8.4 ± 3.8 (2–19)	8.8 ± 4.7 (2–29)	0.285
Numbers of 2PN (mean ± SD)	6.5 ± 3.0 (2–15)	7.0 ± 3.5 (2–20)	0.173

The chi-square (χ^2^) test and Wilcoxon signed rank test were performed to analyze statistical significance. Values in the parentheses were presented as minimal-maximal values or ratio.

### Differences in embryological and ploidy outcomes between biphasic O_2_ (5–2%) and monophasic O_2_ (5%) groups

Paired comparisons revealed no significant differences in the total number of blastocysts between the groups (2.9 ± 1.7 vs 3.8 ± 2.4, Table 2). The number of QBs in the biphasic O_2_ (5–2%) group (2.5 ± 1.6) was significantly higher than that in the monophasic O_2_ (5%) group (1.5 ± 1.2) (*p* < 0.001). However, the formation rates of blastocysts, QBs, and day-5 QBs were significantly higher in the biphasic O_2_ (5–2%) group (54.8%, 35.8%, and 17.5%, respectively) than in the monophasicO_2_ (5%) group (45.4%, 23.5%, and 10.5%, respectively) (*p* < 0.05). Nine cycles (10%, 9/90) in the monophasic O_2_ (5%) group produced no QBs for biopsy. However, all cycles in the biphasic O_2_ (5–2%) group produced at least one QB for biopsy. A total of 137 and 225 QBs from the monophasic (5%) and biphasic O_2_ (5–2%) groups, respectively, were biopsied and subjected to PGT-A. The rate of euploidy in the biphasic O_2_ (5–2%) group (19.6%, 44/225) was significantly higher than that in the monophasic O_2_ (5%) group (0%, 0/137) (*p* < 0.001). The number of euploid blastocysts in the biphasic O_2_ (5–2%) group (0.5 ± 0.8) was significantly higher than that in the monophasic O_2_ (5%) group (0) (*p* < 0.001). The number and formation rate of transferable blastocysts in the biphasic O_2_ (5–2%) group (1.1 ± 1.4, 45.8%, 103/225) were significantly higher than those in the monophasic O_2_ (5%) group (0.5 ± 0.8, 32.8%, 45/137) (*p* < 0.001 and *p* = 0.015, respectively). After incubation under biphasic O_2_ (5–2%) conditions, the incidence of cycles with at least one euploid blastocyst increased from 0 to 33.3% (30/90). In addition, the incidence of cycles with least one transferable blastocyst increased from 35.6% (32/90) to 57.8% (52/90).

**Table 2:** Embryological outcomes in biphasic O_2_ (5–2%) and monophasic O_2_ (5%) concentration groups.

Parameters	Monophasic (5%) O_2_	Biphasic (5–2%) O_2_	*p* value
Cycles (patients)	90	90	–
Numbers of blastocysts (mean ± SD)	2.9 ± 1.7 (0–8)	3.8 ± 2.4 (1–14)	0.095
Numbers of qualified blastocysts (QBs) (mean ± SD)	1.5 ± 1.2 (0–6)	2.5 ± 1.6 (1–9)	< 0.001
Blastocyst rates (%)	45.4 (264/582)	54.8 (344/628)	0.001
QB rates (%)	23.5 (137/582)	35.8 (225/628)	< 0.001
Rates of day 5 QBs (%)	10.5 (61//582)	17.5 (110/628)	< 0.001
Rates of day 6 QBs (%)	13.1 (76/582)	18.2 (114/628)	0.015
Cycles without QBs (%)	10% (9/90)	0 (0/90)	0.002
*Embryo ploidy*			
Numbers of euploid blastocysts (mean ± SD)	0 (0–0)	0.5 ± 0.8 (0–3)	< 0.001
Numbers of aneuploid blastocysts (mean ± SD)	1.0 ± 1.0 (0–5)	1.4 ± 1.2 (0–5)	0.020
Numbers of mosaic blastocysts (mean ± SD)	0.5 ± 0.8 (0–3)	0.7 ± 1.0 (0–5)	0.209
Numbers of transferable blastocysts (mean ± SD)	0.5 ± 0.8 (0–3)	1.1 ± 1.4 (0–5)	< 0.001
Euploidy rate	0 (0/137)	19.6% (44/225)	< 0.001
Rate of transferable blastocyst (%)	32.8% (45/137)	45.8% (103/225)	0.015
Percentage of cycles with at least one euploid blastocyst (%)	0 (0/90)	33.3 (30/90)	< 0.001
Percentage of cycles with at least one transferable blastocyst (%)	35.6 (32/90)	57.8 (52/90)	0.003

The chi-square (χ^2^) test and Wilcoxon signed rank test were performed to analyze statistical significance. Values in the parentheses were presented as minimal-maximal values or ratio.

QB: qualified blastocyst; BR: blastocyst rate per 2PN.

### Correlation between O_2_ tension and the QB formation rate

Univariate regression analysis was performed to identify the individual variables were associated with the formation rate of QBs (Supplementary Table 1). The results indicated that the formation rate of QBs was significantly correlated with the LH level on the day of hCG injection (odds ratio [OR] 0.950, 95% confidence interval [CI] 0.907–0.995; *p* = 0.031), AMH level (OR: 0.923, 95% CI 0.856–0.996; *p* = 0.040), number of oocytes (OR: 0.973, 95% CI 0.953–0.992; *p* = 0.007), number of MII oocytes (OR: 0.965, 95% CI 0.941–0.990; *p* = 0.007), and O_2_ tension (OR: 1.516, 95% CI 1.295–1.775; *p* < 0.001). Considering the LH level on the day of hCG injection and the number of oocytes, the multivariable regression analysis indicated that the biphasic O_2_ (5–2%) condition was positively correlated with the QB formation rate, as compared with the monophasic O_2_ (5%) condition (adjusted OR: 1.535, 95% CI 1.325–1.777; *p* < 0.001) (Table 3).

**Table 3:** The multivariate regression analysis to determine the correlations between variables and the rate of QBs.

Parameters	B	*p* value	OR	95% CI
LH levels (IU/L)	− 0.036	0.074	0.964	0.926–1.004
Numbers of retrieved oocytes	− 0.034	< 0.001	0.966	0.949–0.984
Biphasic O_2_ (5–2%) culture	0.428	< 0.001	1.535	1.325–1.777
Monophasic O_2 _(5%) culture	0		1	

GEE regression was performed to analyze statistical significance.

B: B-coefficient, OR: Odds Ratio, 95% CI: 95% confidence interval.

### Correlation between O_2_ tension and the probability of cycles with transferable blastocysts

Univariate regression analysis was performed to identify potential variables correlated with cycles with transferable blastocysts (Supplementary Table 2). The results indicated that cycles with transferable blastocysts were significantly correlated with the age of the women (OR: 0.781, 95% CI 0.700–0.871; *p* < 0.001), level of E_2_ (OR: 1.001, 95% CI 1.000–1.001; *p* = 0.003), number of 2PN (OR: 1.136, 95% CI 1.038–1.243; *p* = 0.006), number of QBs (OR: 2.336, 95% CI 1.740–3.136; *p* < 0.001), and O_2_ tension (OR: 2.480, 95% CI 1.478–4.161; *p* = 0.001). Therefore, on the basis of these results, age, E_2_ level on the day of hCG injection, and number of 2PN were selected as confounders for adjustment. As shown in Table 4, multivariable regression analysis revealed that the biphasic O_2_ (5–2%) condition was positively correlated with the probability of cycles with transferable blastocysts, as compared with the monophasic O_2_ (5%) condition (adjusted OR: 3.191, 95% CI 1.638–5.679; *p* < 0.001). In addition, patients were further divided into subgroups with age ≤ 38 years and age > 38 years (Supplementary Table 3). The consistent effects of biphasic O_2_ tension strategy were revealed to significantly improve QB rates (37.4% vs. 26.4% and 35.6% vs. 22.4%), euploid rates (25% vs. 0% and 16.8% vs. 0%), and percentages with cycles with at least one transferable blastocyst (46.2% vs. 0% and 28.1% vs. 0%) in both age groups (Supplementary Table 3).

**Table 4:** The multivariate regression analysis to determine the correlations between variables and the probability of cycles with transferable blastocysts.

Parameters	B	*p* value	OR	95% CI
Women age (years)	− 0.243	< 0.001	0.784	0.684–0.857
E_2_ levels (IU/L)	0.000	0.172	1.000	1.000–1.001
Numbers of 2PN	0.101	0.055	1.107	0.998–1.227
Biphasic O_2_ (5–2%) culture	1.160	< 0.001	3.191	1.638–5.679
Monophasic O_2_ (5%) culture	0		1	

GEE regression was performed to analyze statistical significance.

B: B-coefficient, OR: Odds Ratio, 95% CI: 95% confidence interval.

## Discussion

In this study, we evaluated the effects of biphasic O_2_ (5–2%) cultures on embryonic ploidy and development in women without euploid embryos in a previous cycle with monophasic O_2_ (5%). We discovered that biphasic O_2_ (5–2%) is one of the major factors that improves the development of blastocysts. The results of the biphasic O_2_ (5–2%) culture revealed that (1) the number and proportion of QBs considerably increased, (2) the number of euploid embryos and the number of aneuploid embryos increased and decreased, respectively, and (3) the number of transferable QBs and the probability of cycles with transferable QBs increased. Multivariable regression analysis also revealed that the biphasic O_2_ (5–2%) culture was strongly correlated with an increase in the QB formation rate and the probability of cycles with transferable blastocysts.

Although the consumption of O_2_ substantially increases at compaction^31^, during the phase of blastomere differentiation into TE and ICM, low O_2_ tension promotes the metabolic preference of glucose being the primary energy substrate^31,32^, thereby protecting against excess oxidative stress, which is associated with high metabolic activity^33^. Relatively low metabolic levels are strongly linked to embryonic development^34,35^, and surviving embryos usually exhibit reduced oxidative phosphorylation activity and O_2_ consumption^36^. Compared with less metabolically active embryos, embryos with higher active metabolism exhibit increased ROS levels^37^, excessive DNA damage^38^, and poor embryonic development^37^. In addition to endogenous ROS, in embryonic cultures in vitro, high levels of exogenous ROS are formed from the environment during embryonic culture or manipulation^39^. Previous studies have reported that reducing O_2_ tension under culture conditions may decrease the ROS level and cell apoptosis, thereby improving embryonic development in vitro^5,6^. However, hypoxia promotes the formation of blastocysts in cattle, especially during the post compaction phase^40^. These beneficial effects of the low O_2_ concentration during the development of blastocysts in vitro may be stage-dependent^6^. Previous animal studies have reported that biphasic O_2_ (7–2% or 5–2%) tension promotes the formation and quality of preimplantation blastocysts^9,41,42^. Therefore, we hypothesized that adjusting the biphasic O_2_ concentration for different embryonic stages, depending on the requirements of stage-specific metabolism during cleavage and blastocyst formation, has a positive effect on the in vitro cultivation of human embryos.

Despite the effects of hypoxia on metabolism, studies have indicated that the O_2_ concentration affects the expression of genes in preimplantation embryos^43^. In hypoxic environments, O_2_ tension promotes the development of mouse blastocysts and reduces apoptosis by regulating HIF-2α, thereby increasing the expression of antioxidant enzymes, such as MnSOD, PRDX5, and vascular endothelial growth factor (VEGF)^44^. In bovine and mouse preimplantation embryos, biphasic O_2_ (7–2% or 5–2%) changes the expression of genes involved in embryonic metabolism^9,45,46^. Compared with embryos cultured under monophasic O_2_ (20% or 7%) tension, blastocysts cultured under biphasic O_2_ (7–2%) tension have higher ICM and glucose transporter 1 (GLUT-1), glucose transporter 3 (GLUT-3), and VEGF expression^45^. Unlike monophasic O_2_ (5% or 20%) tension, biphasic O_2_ (5–2%) tension increases the developmental capacity and the activation of the HIF-1α transcription factor and related genes of embryonic development, including H2az, Cdx2, Oct-41, and 16 s rRNA^9^. According to Kaser et al., biphasic O_2_ (5–2%) cultures produce an increased total yield of blastocysts and a large number of usable blastocysts, thereby recapitulating the physiologic O_2_ environment in vivo^6^. Brouillet’s study (2021) has also highlighted the clinical outcomes of biphasic O_2_ (5–2%) culture^47^. Generally, biphasic O_2_ (5–2%) cultures are associated with considerably increased cumulative live birth and total blastocyst and usable blastocyst formation rates^47^. Under biphasic O_2_ conditions, changing the relative abundance of anabolic amino acids and metabolites involved in redox homeostasis and the differential expression level of MUC1 in TE cells may improve the development of human embryos ^6^. Moreover, whole transcriptome analysis of blastocysts revealed that 707 RNAs are differentially expressed depending on the strategy of O_2_ supplementation^47^. These genes are mainly involved in embryonic development, DNA repair, embryonic stem cell pluripotency, and implantation potential^47^. In this study, we confirmed that in patients with a poor prognosis, the rate of blastocyst formation in biphasic O_2_ (5–2%) cultures was higher than that in monophasic O_2_ (5%) cultures (Table 2). We also reported that all cycles in biphasic O_2_ (5–2%) cultures have at least one QB suitable for embryonic biopsy (Table 2).

To the best of our knowledge, this is the first study to examine the effects of biphasic O_2_ (5–2%) tension on blastocyst ploidy. Generally, well-balanced embryonic development is associated with optimal and quiet metabolism^35^,instead of active metabolism, which negatively affects the quality of embryos by increasing the ROS levels^37^. In addition, during in vitro cultivation, ROS from endogenous and exogenous sources may result in molecular damage to embryonic chromosome segregation apparatuses and may cause aneuploidy over time^48^. In mouse embryos obtained through IVF, ROS-induced damage seems to increase the incidence of chromosomal aneuploidy^16^. During embryonic development, ROS overproduction is associated with negative outcomes, such as impaired mitochondrial function, aging, DNA damage, and impaired chromosome segregation^49^. Therefore, reducing O_2_ tension under culture conditions has been suggested to improve the genomic integrity of developing embryos by decreasing the levels of ROS^5,6^.

Our results revealed that the patients included in this study (with a poor prognosis) had a considerably increased number of euploid blastocysts after biphasic O_2_ (5–2%) culture (Table 2). After incubation was performed under a biphasic O_2_ (5–2%) condition, the incidence of cycles with at least one euploid blastocyst increased from 0 to 33.3% (30/90) (Table 2). Because the patients had no or a small number of euploid blastocysts suitable for transfer, they were presented with the option of mosaic blastocyst transfer. According to previous studies, mosaic embryos may result in healthy babies^50,51^. In this study, all euploid and mosaic embryos are defined as transferable blastocysts, the number of transferable blastocysts and the probability of cycles with transferable blastocysts substantially increased under the biphasic O_2_ (5–2%) condition compared with under the monophasic O_2_ (5%) condition (Table 4). Given the age of the women included in this study, biphasic O_2_ (5–2%) tension still increased not only the number of euploid blastocysts but also the rate of euploid blastocyst formation. Studies have indicated that self-correction of aneuploid and mosaic embryos considerably increases after the eight-cell stage^52^, and that approximately 9.7% of day-3 aneuploid embryos undergo complete self-correction to form euploid blastocysts^52^. Therefore, we suggest that an ultralow O_2_ (2%) concentration of the biphasic O_2_ (5–2%) strategy may further improve self-correction during blastocyst formation by reducing the effects of ROS.

In addition, women's age was also related to ploidy status in this study. The spearman's correlation (Supplementary Table 4) analysis showed that women age significantly negatively correlation with euploidy (r: − 0.189, *p* < 0.05) and positively correlation with aneuploidy (r: 0.437, *p* < 0.01) rates. The multivariable regression analysis revealed that the women age was negatively correlated with the probability of cycles with transferable blastocysts (adjusted OR: 0.784, 95% CI 0.684–0.857; *p* < 0.001; Table 4) after adjustment of O_2_ conditions. This study demonstrated that even in the biphasic O_2_ (5–2%) culture condition, women age remained an important factor associated with embryo ploidy. The increase of euploid rate (from 0 to 25%) in the younger patient group was higher than that (from 0% to 16.8%) in the older patient group (Supplementary Table 3). Previous studies have reported that increased ROS and elevated vulnerability of oocytes to ROS lead to reduce the fertilization and developmental competency in aged patients^53,54^. Therefore, the beneficial effects of biphasic O_2_ (5–2%) culture might be influenced by the insufficient antioxidant capability in older patients^55^.

Because the oocytes derived from one stimulation cycle could not be divided into different groups in some of the target patients, we compared the effects of biphasic O_2_ (5–2%) and monophasic O_2_ (5%) cultures by using a design of self-paired comparison from two continuous cycles of individual patients. However, one limitation must be acknowledged; that is, we discovered that a few baseline characteristics differed between cycles (i.e., number of retrieved oocytes) (Table 1). Nevertheless, the numbers of mature oocytes and 2PN were similar between the two groups, and Spearman’s correlation analysis revealed that the baseline characteristics were not correlated with the primary and secondary outcomes (Table 1 and Supplementary Table 4). Therefore, by considering confounders, we further confirmed the beneficial effects of biphasic O_2_ (5–2%) cultures on the rate of QBs formation and the probability of cycles with transferable blastocysts. In general, embryos cultured at reduced O_2_ levels from 20 to 2% require a nitrogen gas system and quality control procedures for the O_2_ sensors. Compared with the atmospheric or monophasic O_2_ (5%) culture conditions adopted by the majority of embryological laboratories, biphasic O_2_ (5–2%) cultures may result in increased laboratory equipment costs. Moreover, given the nature of retrospective studies, which lack randomization and may involve selection bias, randomized controlled trials are suggested to robustly assess these interesting results.

In this study, we included women who had a small number of QBs and lacked euploid blastocyst formation under monophasic O_2_ (5%) culture conditions. The results revealed that biphasic O_2_ (5–2%) culture not only improved the development of QBs but also promoted the formation of euploid blastocysts, thereby overcoming the challenges of embryonic development. This technique also considerably increased the probability of cycles with transferable blastocysts. In conclusion, biphasic O_2_ (5–2%) culture can be used as an alternative strategy to increase the incidence of transferable blastocysts and to reduce the cancellation rate of blastocyst transfer in patients with a poor prognosis.

## Materials and methods

### Patient selection

This was a retrospective cohort study and a single-center clinical trial that aimed to evaluate the effects of O_2_ tension, including monophasic O_2_ (5%) and biphasic O_2_ (5–2%) culture systems, on blastocyst formation and ploidy in PGT-A cycles. The study cohort included 90 women with 180 cycles who were referred to Lee Women’s Hospital, Taiwan, and treated with PGT-A from March 2018 to March 2021. Patients who had no euploid blastocysts in monophasic O_2_ (5%) cultures in previous cycles and underwent biphasic O_2_ (5–2%) cultures in the following cycles were included, whereas oocyte and sperm donors and patients with male factors, such as sperms obtained from testicular or epididymal sperm aspiration or extraction, were excluded. PGT-A cycles were divided into two subgroups of embryo culture, namely monophasic O_2_ (5%) and biphasic O_2_ (5–2%). The treatment histories and clinical outcomes of all patients were recorded from the database of Lee Women’s Hospital. This retrospective data analysis was approved by the Institutional Review Board of Chung Shan Medical University, Taichung, Taiwan (CS1-21156). Our study methods and analysis conform to the guidelines and regulations set by the agreement with the Institutional Review Board of Chung Shan Medical University IRB. The need to obtain informed consent in this study was waived by the Institutional Review Board of Chung Shan Medical University IRB.

### Controlled ovarian stimulation protocols

Both gonadotropin-releasing hormone (GnRH) agonist (Lupron; Takeda Chemical Industries, Tokyo, Japan) and antagonist (cetrorelix acetate; Merck Serono, Darmstadt, Germany) protocols were used for controlled ovarian stimulation (COS). For ovarian stimulation, all patients received a recombinant follicle-stimulating hormone (FSH, Gonal-F; Merck Serono) from cycle day 3 until the diameter of the dominant follicle exceeded 18 mm, followed by a dual trigger, including 250 μg of human chorionic gonadotropin (hCG) (Ovidrel; EMD Serono, Rockland, MA, USA), and 0.2 mg of triptorelin (Decapeptyl; Ferring, Schleswig–Holstein, Germany) at 36 h before oocyte retrieval. Finally, COS procedures involving GnRH agonist and antagonist protocols, oocyte collection, and denudation were performed as previously described^56^.

### Embryo culture

Before fertilization, the retrieved oocytes were cultured in Quinn’s Advantage Fertilization Medium (Sage BioPharma, Trumbull, CT, USA) supplemented with 15% serum protein substitute (SPS; Sage BioPharma) in a triple gas phase under 5% CO_2_, 5% O_2_, and 90% N_2_. The insemination methods in this study were including ICSI (110 cycles) and half-ICSI (70 cycles, oocytes from the same patient are divided into half of the oocytes for conventional insemination (IVF) and the other half for ICSI). Conventional insemination (IVF) or intracytoplasmic sperm injection (ICSI) was then performed 38–41 h after the dual trigger. Following conventional insemination or ICSI, all embryos were further cultured in microdrops of cleavage medium (Quinn’s Advantage Cleavage Medium; Sage BioPharma) supplemented with 15% SPS. Embryo culture was then performed until day 3 (at 70–72 h after insemination or ICSI) in cleavage medium under 5% O_2_. On day 3, all cleaved embryos were group-cultured in microdrops of blastocyst medium (Quinn’s Advantage Blastocyst Medium; Sage BioPharma) supplemented with 15% SPS. The culture systems were divided into monophasic O_2_ (5%) and biphasic O_2_ (5–2%) tension groups. In the monophasic O_2_ (5%) tension group, the embryos were cultured in a 5% O_2_ incubator until day 5/6. In the biphasic O_2_ (5–2%) tension group, the embryos were transferred into 2% O_2_ incubators on day 3 for culturing until day 5/6. All embryos were individually cultured before day 3 and then group-cultured thereafter. Laser-assisted hatching was performed on day 4. Once the embryos reached the blastocyst stage, trophectoderm (TE) biopsy was performed as described by Chen et al.^56^. The quality of the blastocysts was then immediately assessed before TE biopsy according to the criteria of Gardner and Schoolcraft^57^. Only high-quality expanded blastocysts (grade 4, 5, 6, AB, BA, or BB) with a blastocyst diameter ≥ 150 μm were defined as QBs suitable for TE biopsy. On day 5, fully expanded or hatching blastocysts with a qualified TE and inner cell mass (ICM) were considered adequate for TE biopsy. Blastocysts without expansion (< 150 μm) on day 5 were further cultured until day 6, and TE biopsy was then performed on the expanded blastocysts with ICM and TE grade A or B. The biopsied TE cells were then immediately placed in an RNAse/DNAse-free polymerase chain reaction tube and amplified using a SurePlex DNA Amplification System (Illumina, San Diego, CA, USA). The extracted cells were then added to 2 μL of buffer and shipped frozen to the genetics laboratory (Genesis Genetics) for PGT-A on a high-resolution next-generation sequencing platform. After TE biopsy, the blastocysts were cryopreserved through vitrification conducted by Cryotech^56^. According to the next-generation sequencing results, blastocysts without aneuploidy were defined as "transferable blastocysts", and cycles with least one transferable blastocyst were defined as "cycles with transferable blastocysts".

### Statistical analysis

Wilcoxon’s signed-rank test was used to perform pair comparisons of nonparametric and count data. The chi-squared test was then used to compare the percentage results of the biphasic O_2_ (5–2%) and monophasic O_2_ (5%) groups. On the basis of Spearman rank correlation coefficients, the variables were analyzed through a generalized estimating equation (GEE) and univariable regression was conducted. The outcome variables included the probability of cycles with transferable blastocysts (yes/no) and predictors such as the age of the women (per year), the total FSH dosage (per 1,000 IU), the number of retrieved oocytes (per number), the number of MII oocytes (per number), the number of 2PN (per number), the blastocyst formation rate (per 10%), the insemination method (ICSI/half ICSI), and the O_2_ tension type (biphasic/monophasic O_2_ tension). Logistic and Poisson’s regression analyses were then performed to evaluate the effect of confounding factors on binary data representing the probability of cycles with transferable blastocysts and the count data of QBs, respectively. The total number of 2PN per cycle served as the offset for the Poisson regression model, and a difference with *p* < 0.05 was regarded as statistically significant. All calculations were performed using SPSS version 23.0 (IBM SPSS, Armonk, NY).

## Supplementary Information

Supplementary Information 1.

Supplementary Information 2.

Supplementary Information 3.

Supplementary Information 4.

## Data Availability

The data underlying this article will be shared on reasonable request to the corresponding author.

## References

[1] Byatt-Smith JG, Leese HJ, Gosden RG (1991). An investigation by mathematical modelling of whether mouse and human preimplantation embryos in static culture can satisfy their demands for oxygen by diffusion. Hum. Reprod..

[2] Fischer B, Bavister BD (1993). Oxygen tension in the oviduct and uterus of rhesus monkeys, hamsters and rabbits. J. Reprod. Fertil..

[3] Kasterstein E (2013). The effect of two distinct levels of oxygen concentration on embryo development in a sibling oocyte study. J. Assist. Reprod. Genet..

[4] Li J, Foote RH, Simkin M (1993). Development of rabbit zygotes cultured in protein-free medium with catalase, taurine, or superoxide dismutase. Biol. Reprod..

[5] Yang Y (2016). Comparison of 2, 5, and 20% O_2_ on the development of post-thaw human embryos. J. Assist. Reprod. Genet..

[6] Kaser DJ (2018). Randomized controlled trial of low (5%) versus ultralow (2%) oxygen for extended culture using bipronucleate and tripronucleate human preimplantation embryos. Fertil. Steril..

[7] Morin SJ (2017). Oxygen tension in embryo culture: does a shift to 2% O_2_ in extended culture represent the most physiologic system?. J. Assist. Reprod. Genet..

[8] Ottosen LD (2006). Observations on intrauterine oxygen tension measured by fibre-optic microsensors. Reprod. Biomed. Online.

[9] Choi J, Kim W, Yoon H, Lee J, Jun JH (2021). Dynamic oxygen conditions promote the translocation of HIF-1α to the nucleus in mouse blastocysts. Biomed. Res. Int..

[10] Okazaki K, Maltepe E (2006). Oxygen, epigenetics and stem cell fate. Regen. Med..

[11] Lee SC, Seo HC, Lee J, Jun JH, Choi KW (2019). Effects of dynamic oxygen concentrations on the development of mouse pre- and peri-implantation embryos using a double-channel gas supply incubator system. Clin. Exp. Reprod. Med..

[12] Li M, Xue X, Shi J (2022). Ultralow oxygen tension (2%) is beneficial for blastocyst formation of in vitro human low-quality embryo culture. Biomed. Res. Int..

[13] Belli M (2019). Oxygen concentration alters mitochondrial structure and function in in vitro fertilized preimplantation mouse embryos. Hum. Reprod..

[14] Bean CJ, Hassold TJ, Judis L, Hunt PA (2002). Fertilization in vitro increases non-disjunction during early cleavage divisions in a mouse model system. Hum. Reprod..

[15] Katz-Jaffe M, Parks J, McReynolds S, Henry L, Schoolcraft WB (2018). Chromosomal mosaicism is impacted by compromised embryo culture conditions. Fertil. Steril..

[16] Huang Y, Ha S, Li Z, Li J, Xiao W (2019). CHK1-CENP B/MAD2 is associated with mild oxidative damage-induced sex chromosome aneuploidy of male mouse embryos during in vitro fertilization. Free Radic. Biol. Med..

[17] Munne S, Wells D (2017). Detection of mosaicism at blastocyst stage with the use of high-resolution next-generation sequencing. Fertil. Steril..

[18] Liochev SI (2013). Reactive oxygen species and the free radical theory of aging. Free Radic. Biol. Med..

[19] Shih YF (2014). Effects of reactive oxygen species levels in prepared culture media on embryo development: A comparison of two media. Taiwan J. Obstet. Gynecol..

[20] Formella I (2018). Real-time visualization of oxidative stress-mediated neurodegeneration of individual spinal motor neurons in vivo. Redox Biol..

[21] Nguyen AL (2017). Identification and characterization of Aurora kinase B and C variants associated with maternal aneuploidy. Mol. Hum. Reprod..

[22] Anderson A, Graff K, Crain J (2007). Low oxygen tension and euploidy after preimplantation genetic screening. Fertil. Steril..

[23] Pagidas K, Ying Y, Keefe D (2008). Predictive value of preimplantation genetic diagnosis for aneuploidy screening in repeated IVF-ET cycles among women with recurrent implantation failure. J. Assist. Reprod. Genet..

[24] Simon AL (2018). Pregnancy outcomes from more than 1,800 in vitro fertilization cycles with the use of 24-chromosome single-nucleotide polymorphism-based preimplantation genetic testing for aneuploidy. Fertil. Steril..

[25] Murugappan G, Shahine LK, Perfetto CO, Hickok LR, Lathi RB (2016). Intent to treat analysis of in vitro fertilization and preimplantation genetic screening versus expectant management in patients with recurrent pregnancy loss. Hum. Reprod..

[26] Harper J (2010). What next for preimplantation genetic screening (PGS)? A position statement from the ESHRE PGD Consortium steering committee. Hum. Reprod..

[27] Wilton L (2002). Preimplantation genetic diagnosis for aneuploidy screening in early human embryos: a review. Prenat. Diagn..

[28] Rubio C (2005). Embryo aneuploidy screening for unexplained recurrent miscarriage: A minireview. Am. J. Reprod. Immunol..

[29] Haddad G (2015). Assessment of aneuploidy formation in human blastocysts resulting from donated eggs and the necessity of the embryos for aneuploidy screening. J. Assist. Reprod. Genet..

[30] Franasiak JM (2014). Aneuploidy across individual chromosomes at the embryonic level in trophectoderm biopsies: changes with patient age and chromosome structure. J. Assist. Reprod. Genet..

[31] Leese HJ (1995). Metabolic control during preimplantation mammalian development. Hum. Reprod. Update.

[32] Gott AL, Hardy K, Winston RM, Leese HJ (1990). Non-invasive measurement of pyruvate and glucose uptake and lactate production by single human preimplantation embryos. Hum. Reprod..

[33] Guerin P, El Mouatassim S, Menezo Y (2001). Oxidative stress and protection against reactive oxygen species in the pre-implantation embryo and its surroundings. Hum. Reprod. Update.

[34] Leese HJ (2002). Quiet please, do not disturb: a hypothesis of embryo metabolism and viability. BioEssays.

[35] Lin E, Li Z, Huang Y, Ru G, He P (2020). High dosages of equine chorionic gonadotropin exert adverse effects on the developmental competence of IVF-derived mouse embryos and cause oxidative stress-induced aneuploidy. Front. Cell Dev. Biol..

[36] Madrid Gaviria S (2019). Resveratrol supplementation promotes recovery of lower oxidative metabolism after vitrification and warming of in vitro-produced bovine embryos. Reprod. Fertil. Dev..

[37] Leese HJ, Sturmey RG, Baumann CG, McEvoy TG (2007). Embryo viability and metabolism: obeying the quiet rules. Hum. Reprod..

[38] Baumann CG, Morris DG, Sreenan JM, Leese HJ (2007). The quiet embryo hypothesis: molecular characteristics favoring viability. Mol. Reprod. Dev..

[39] Cebral E, Carrasco I, Vantman D, Smith R (2007). Preimplantation embryotoxicity after mouse embryo exposition to reactive oxygen species. Biocell.

[40] Thompson JG, Peterson AJ (2000). Bovine embryo culture in vitro: new developments and post-transfer consequences. Hum. Reprod..

[41] Harvey AJ, Kind KL, Pantaleon M, Armstrong DT, Thompson JG (2004). Oxygen-regulated gene expression in bovine blastocysts. Biol. Reprod..

[42] Nguyen AQ (2020). Mouse embryos exposed to oxygen concentrations that mimic changes in the oviduct and uterus show improvement in blastocyst rate, blastocyst size, and accelerated cell division. Reprod. Biol..

[43] Rinaudo PF, Giritharan G, Talbi S, Dobson AT, Schultz RM (2006). Effects of oxygen tension on gene expression in preimplantation mouse embryos. Fertil. Steril..

[44] Ma YY, Chen HW, Tzeng CR (2017). Low oxygen tension increases mitochondrial membrane potential and enhances expression of antioxidant genes and implantation protein of mouse blastocyst cultured in vitro. J. Ovarian Res..

[45] Kind KL, Collett RA, Harvey AJ, Thompson JG (2005). Oxygen-regulated expression of GLUT-1, GLUT-3, and VEGF in the mouse blastocyst. Mol. Reprod. Dev..

[46] Harvey AJ (2007). Differential expression of oxygen-regulated genes in bovine blastocysts. Mol. Reprod. Dev..

[47] Brouillet S (2021). Biphasic (5–2%) oxygen concentration strategy significantly improves the usable blastocyst and cumulative live birth rates in in vitro fertilization. Sci. Rep..

[48] Sasaki H (2019). Impact of Oxidative Stress on Age-Associated Decline in Oocyte Developmental Competence. Front Endocrinol (Lausanne).

[49] Aitken RJ (2020). Impact of oxidative stress on male and female germ cells: implications for fertility. Reproduction.

[50] Lin PY (2020). Clinical Outcomes of single mosaic embryo transfer: high-level or low-level mosaic embryo, does it matter?. J. Clin. Med..

[51] Lee CI (2020). Healthy live births from transfer of low-mosaicism embryos after preimplantation genetic testing for aneuploidy. J. Assist. Reprod. Genet..

[52] Barbash-Hazan S (2009). Preimplantation aneuploid embryos undergo self-correction in correlation with their developmental potential. Fertil. Steril..

[53] Igarashi H, Takahashi T, Nagase S (2015). Oocyte aging underlies female reproductive aging: biological mechanisms and therapeutic strategies. Reprod. Med. Biol..

[54] Thouas GA, Trounson AO, Jones GM (2005). Effect of female age on mouse oocyte developmental competence following mitochondrial injury. Biol. Reprod..

[55] Hamatani T (2004). Age-associated alteration of gene expression patterns in mouse oocytes. Hum. Mol. Genet..

[56] Chen HH (2017). Optimal timing of blastocyst vitrification after trophectoderm biopsy for preimplantation genetic screening. PLoS ONE.

[57] Gardner DK, Schoolcraft WB (1999). Culture and transfer of human blastocysts. Curr. Opin. Obstet. Gynecol..

