# Supplementary material for: Biphasic oxygen tension promotes the formation of transferable blastocysts in patients without euploid embryos in previous monophasic oxygen cycles
Source: Sci Rep. 2023 Mar 15;13:4330. doi: 10.1038/s41598-023-31472-4 (PMC10017668; doi:10.1038/s41598-023-31472-4)
Supplement: Supplementary file 1 — Supplementary Information 1. [file 41598_2023_31472_MOESM1_ESM.docx]

Supplementary Table 1. The univariate regression analysis to determine the correlations between variables and the rate of QBs

|  | B | p value | OR | 95% CI |
| --- | --- | --- | --- | --- |
| Women age (years) | -0.012 | 0.415 | 0.988 | 0.959-1.018 |
| Duration of infertility (years) | -0.008 | 0.528 | 0.992 | 0.966-1.018 |
| Body mass index (BMI, Kg/m2) | -0.013 | 0.512 | 0.987 | 0.949-1.026 |
| Anti-Mullerian Hormone (AMH, ng/mL) | -0.080 | 0.040 | 0.923 | 0.856-0.996 |
| GnRH antagonist | 0.232 | 0.139 | 1.261 | 0.927-1.716 |
| GnRH agonist | 0 |  | 1 |  |
| FSH dosage | 4.807E-05 | 0.555 | 1.000 | 1.000-1.000 |
| LH levels (IU/L) | -0.051 | 0.031 | 0.950 | 0.907-0.995 |
| E_2_ levels (IU/L) | -3.041E-05 | 0.694 | 1.000 | 1.000-1.000 |
| P_4_ levels (ng/mL) | -0.081 | 0.524 | 0.922 | 0.720-1.182 |
| Numbers of retrieved oocytes | -0.028 | 0.007 | 0.973 | 0.953-0.992 |
| Numbers of MII oocytes | -0.035 | 0.007 | 0.965 | 0.941-0.990 |
| Half-ICSI | -0.042 | 0.701 | 0.959 | 0.773-1.189 |
| ICSI | 0 |  | 1 |  |
| Biphasic O2 (5%-2%) culture | 0.416 | 0.000 | 1.516 | 1.295-1.775 |
| Monophasic O2 (5%) culture | 0 |  | 1 |  |

B: B-coefficient, OR: Odds Ratio, 95% CI: 95% confidence interval; Half-ICSI: the insemination methods combined with conventional insemination (IVF) and ICSI

GEE regression was performed to analyze statistical significance.
