# Supplementary material for: Biphasic oxygen tension promotes the formation of transferable blastocysts in patients without euploid embryos in previous monophasic oxygen cycles
Source: Sci Rep. 2023 Mar 15;13:4330. doi: 10.1038/s41598-023-31472-4 (PMC10017668; doi:10.1038/s41598-023-31472-4)
Supplement: Supplementary file 2 — Supplementary Information 2. [file 41598_2023_31472_MOESM2_ESM.docx]

Supplementary Table 2. The univariate regression analysis to determine the correlations between variables and the probability of cycles with transferable blastocysts

|  | B | p value | OR | 95% CI |
| --- | --- | --- | --- | --- |
| Women age (years) | -0.248 | 0.000 | 0.781 | 0.700-0.871 |
| Duration of infertility (years) | -0.018 | 0.672 | 0.982 | 0.903-1.068 |
| Body mass index (BMI, Kg/m^2^) | 0.061 | 0.191 | 1.063 | 0.970-1.164 |
| Anti-Mullerian Hormone (AMH, ng/mL) | 0.012 | 0.864 | 1.012 | 0.884-1.158 |
| GnRH antagonist | -0.437 | 0.319 | 0.646 | 0.273-1.526 |
| GnRH agonist | 0 |  | 1 |  |
| FSH dosage | 0.000 | 0.410 | 1.000 | 1.000-1.001 |
| LH levels (IU/L) | -0.035 | 0.601 | 0.965 | 0.846-1.102 |
| E_2_ levels (IU/L) | 0.001 | 0.003 | 1.001 | 1.000-1.001 |
| P_4_ levels (ng/mL) | 0.091 | 0.735 | 1.095 | 0.646-1.857 |
| Numbers of retrieved oocytes | 0.052 | 0.092 | 1.053 | 0.992-1.119 |
| Numbers of MII oocytes | 0.068 | 0.057 | 1.070 | 0.998-1.148 |
| Biphasic O_2_ (5%-2%) culture | 0.908 | 0.001 | 2.480 | 1.478-4.161 |
| Monophasic O_2_ (5%) culture | 0 |  | 1 |  |
| Half-ICSI | 0.563 | 0.091 | 1.756 | 0.913-3.378 |
| ICSI | 0 |  | 1 |  |
| Numbers of 2PN | 0.127 | 0.006 | 1.136 | 1.038-1.243 |
| Numbers of QBs | 0.848 | <0.001 | 2.336 | 1.740-3.1636 |

B: B-coefficient, OR: Odds Ratio, 95% CI: 95% confidence interval, Half-ICSI: the insemination methods combined with conventional insemination (IVF) and ICSI

GEE regression was performed to analyze statistical significance.
