# Supplementary material for: Biphasic oxygen tension promotes the formation of transferable blastocysts in patients without euploid embryos in previous monophasic oxygen cycles
Source: Sci Rep. 2023 Mar 15;13:4330. doi: 10.1038/s41598-023-31472-4 (PMC10017668; doi:10.1038/s41598-023-31472-4)
Supplement: Supplementary file 3 — Supplementary Information 3. [file 41598_2023_31472_MOESM3_ESM.docx]

Supplementary Table 3. Embryological outcomes in different women age and O_2_ concentration groups

| **Women age (years)** | **≤ 38** | |  | **> 38** | | |  |
| --- | --- | --- | --- | --- | --- | --- | --- |
| **O_2_ tension group** | **Monophasic O_2_ (5%)** | **Biphasic O_2_ (5–2%)** | **p values** | **Monophasic O_2_ (5%)** | | **Biphasic O_2_ (5–2%)** | **p values** |
| Cycles (patients) | 26 | 26 | - | 64 | 64 | | - |
| Numbers of blastocysts (mean ± SD) | 2.8 ± 1.7 | 4.3 ± 3.1 | 0.005 | 3.0 ± 1.7 | 3.6 ± 2.1 | | 0.026 |
| Numbers of qualified blastocysts (QBs) (mean ± SD) | 1.7 ± 1.2 | 2.9 ± 2.0 | 0.001 | 1.5 ± 1.2 | 2.3 ± 1.3 | | <0.001 |
| Blastocyst rates (%) | 45.4% (74/163) | 55.7% (113/203) | 0.050 | 45.3% (190/395) | 55.1% (231/419) | | 0.005 |
| QB rates (%) | 26.4% (43/163) | 37.4% (76/203) | 0.026 | 22.4% (94/395) | 35.6% (149/419) | | <0.001 |
| Rates of day 5 QBs (%) | 39.5% (17/43) | 50% (38/76) | 0.300 | 46.8% (44/190) | 48.3% (72/149) | | 0.820 |
| Rates of day 6 QBs (%) | 60.5% (26/43) | 48.7% (37/76) | 0.216 | 53.2% (50/190) | 51.7% (77/149) | | 0.820 |
| Cycles without QBs (%) | 3.8% (1/26) | 0% (0/26) | 1.000 | 12.5% (8/64) | 0% (0/64) | | 0.003 |
| **Embryo ploidy** |  |  |  |  |  | |  |
| Numbers of euploid blastocysts (mean ± SD) | 0 | 0.7 ± 1.0 | 0.002 | 0 | 0.4 ± 0.7 | | <0.001 |
| Numbers of aneuploid blastocysts (mean ± SD) | 0.9 ± 1.1 | 0.9 ± 1.2 | 0.704 | 1.1 ± 1.0 | 1.5 ± 1.1 | | 0.007 |
| Numbers of mosaic blastocysts (mean ± SD) | 0.7 ± 0.9 | 1.3 ± 1.2 | 0.044 | 0.4 ± 0.8 | 0.4 ± 0.7 | | 0.946 |
| Numbers of transferable blastocysts (mean ± SD) | 0.7 ± 0.9 | 2.0 ± 1.6 | 0.001 | 0.4 ± 0.8 | 0.8 ± 1.2 | | 0.007 |
| Euploidy rate | 0% (0/43) | 25% (19/76) | <0.001 | 0% (0/190) | 16.8% (25/149) | | <0.001 |
| Rate of transferable blastocyst (%) | 44.2% (19/43) | 69.7% (53/76) | 0.006 | 27.7% (26/94) | 33.6% (50/149) | | 0.334 |
| Percentage of cycles with at least one euploid blastocyst (%) | 53.8% (14/26) | 84.6% (22/26) | 0.016 | 28.1% (18/64) | 46.9% (30/64) | | 0.028 |
| Percentage of cycles with at least one transferable blastocyst (%) | 0% (0/26) | 46.2% (12/26) | <0.001 | 0% (0/64) | 28.1% (18/64) | | <0.001 |

QBs: qualified blastocysts; BR: blastocyst rate per 2PN.

The chi-square (χ ^2^) test and Wilcoxon signed rank testwere performed to analyze statistical significance.
