# Supplementary material for: Biphasic oxygen tension promotes the formation of transferable blastocysts in patients without euploid embryos in previous monophasic oxygen cycles
Source: Sci Rep. 2023 Mar 15;13:4330. doi: 10.1038/s41598-023-31472-4 (PMC10017668; doi:10.1038/s41598-023-31472-4)
Supplement: Supplementary file 4 — Supplementary Information 4. [file 41598_2023_31472_MOESM4_ESM.docx]

Supplementary Table 4. Spearman’s correlation matrix of associations between selected variables of embryo development and basic parameters

half-ICSI: the insemination methods combined with conventional insemination (IVF) and ICSI.

Spearman’s correlation coefficients (*r*) between evaluated parameters are presented. A p-value of < 0.05 was considered statistically significant (* p < 0.05; ** p < 0.01; *** p < 0.001).
